# Welander Distal Myopathy-Associated TIA1 E384K Mutation Disrupts Stress Granule Dynamics Under Distinct Stress Conditions

**DOI:** 10.3390/biology14091288

**Published:** 2025-09-18

**Authors:** Beatriz Ramos-Velasco, José Alcalde, José M. Izquierdo

**Affiliations:** Centro de Biología Molecular Severo Ochoa (CBM), Agencia Estatal Consejo Superior de Investigaciones Científicas (CSIC), Universidad Autónoma de Madrid (UAM), C/Nicolás Cabrera 1, Cantoblanco, 28049 Madrid, Spain; beatriz.ramosvel@gmail.com (B.R.-V.); jalcalde@cbm.csic.es (J.A.)

**Keywords:** TIA1, stress granules, Welander distal myopathy, heat shock, proteostatic insult, oxidative stress, osmotic challenge

## Abstract

Cellular stress triggers the formation of diverse RNA–protein aggregates, which can be associated with physiological responses, pathological conditions, or even detrimental outcomes. Under stress-induced proteostasis disruption, these RNA–protein assemblies are known as stress granules (SGs). Targeting such condensates—while sparing functional RNAs and proteins—remains a major therapeutic challenge in protein aggregation disorders such as myopathies and neuropathies. In this study, we investigated the cellular response to various stress conditions in the context of the TIA1 E384K mutation, a founder variant implicated in both Welander distal myopathy (WDM) and amyotrophic lateral sclerosis (ALS). Cells were exposed to different stressors, including proteotoxic, proteostatic, chemotoxic, and osmotic insults, and the behavior of TIA1-related SGs was analyzed. Our findings reveal a distinct yet conserved pattern in the dynamics of TIA1-dependent SG formation and clearance, influenced by the specific type of stressor and modulated by eIF2α Ser35 phosphorylation. These results indicate that the WDM-associated TIA1 mutation leads to aberrant SG dynamics across different stress conditions. Collectively, these observations support the idea that TIA1 E384K-associated SG dysregulation plays a role in WDM and ALS pathogenesis and underscores the importance of multiple stress contexts in disease progression.

## Simple Summary

Welander distal myopathy (WDM; OMIM: 604454) is a rare distal dystrophy characterized by weakness in the distal upper extremities, usually finger and wrist extensors, which later progresses to all hand muscles and distal lower extremities, primarily in toe and ankle extensors. This disorder remains poorly investigated and underdocumented from clinical and biomedical perspectives. WDM is caused by a missense change (c.1362G > A; p.E384K) in the T-cell intracellular antigen 1 (*TIA1*) gene. TIA1 is a ubiquitously expressed multifunctional RNA-binding protein that plays a key role in the metabolism and fate of cellular proteins and RNAs. TIA1 is a master regulator of gene expression and impacts the cellular responses involved in stress conditions. A prevalent regulatory aspect directly mediated by TIA1 has been linked to WDM: the dynamics of TIA1-dependent stress granules (SGs), which play a protective/survival function in eukaryotes in response to environmental stress. Our previous findings confirmed and expanded on the deleterious effects of TIA1^WDM^ on SG dynamics. Our research fully aligns with both the acquisition of knowledge and the search for therapeutic solutions to document and advance all areas related to this pathology to improve the health and quality of life of patients and their families.

## 1. Introduction

Eukaryotic cells and other biological systems exhibit dynamic responses—characterized by versatility, flexibility, and speed—to fluctuating internal and external conditions. Central to this transient adaptation is the control and modulation of gene expression, which critically influences cell fate and survival. Specifically, cellular responses to intra- and/or extracellular changes primarily rely on post-transcriptional regulation of gene expression. This involves processes such as the splicing of pre-mRNAs, RNA transport, mRNA stability, and translation, resulting in targeted reshaping of both the cellular transcriptome and proteome. RNA-binding proteins (RBPs) and other non-RBPs serve as key orchestrators of these molecular events, driving regulatory reprogramming. They work in concert with diverse RNA populations, including protein-coding RNAs (mRNAs), short and long non-coding RNAs (ncRNAs), microRNAs (miRNAs), and circular RNAs (circRNAs), to coordinate and execute the complex processes of post-transcriptional gene regulation [1].

Cellular stress poses a severe threat to cell survival. Through evolution, eukaryotic cells have developed adaptive mechanisms and processes to navigate these critical challenges. A notable example is the formation of stress granules (SGs) [2,3]. These are heterogeneous, non-membrane structures with a liquid or gel-like consistency and properties distinct from those of the surrounding cytoplasm driven by the liquid–liquid phase separation (LLPS) phenomenon [4,5,6,7,8,9,10]. The intricate dynamics of SG assembly and disassembly, governed by complex biophysical processes, remain only partially understood [3,5]. These structures are heterogeneous aggregates comprising various proteins, such as RBPs, non-RBPs, eukaryotic translation initiation factors, and translation-unrelated proteins, alongside diverse RNA molecules, including mRNAs, ncRNAs, miRNAs, and circRNAs. The clustering of these components within SGs results from intricate protein–protein, RNA-RNA, and protein–RNA interactions [2,3,4,5,6,7,8,9,10].

T-cell intracellular antigen 1 (TIA1) is a multifunctional RBP that plays a key regulatory role across multiple layers of gene expression [11,12,13,14,15]. Its activity is central to various biological processes, impacting the physiological and pathological functions of cells, tissues, and organisms. Notably, TIA1 is a core component of post-transcriptional gene regulation and is critically involved in SG dynamics, including their formation, assembly, and disassembly [4,5,6,7,8,9,10,15,16,17,18].

Welander distal myopathy (WDM) is a rare autosomal dominant muscular dystrophy that predominantly affects individuals of northern European descent, particularly in Sweden and parts of Finland [19]. The disease typically manifests in adulthood, between the ages of 40 and 60, with early symptoms including weakness in the thumb and/or index finger. This weakness gradually impairs the extension and functionality of the remaining fingers, ultimately affecting fine motor skills and the intrinsic muscles of the hand. As the disease progresses, distal muscles of the lower limbs also become involved, leading to gait disturbances and reduced mobility. Histopathological examination reveals characteristic myopathic changes, including filamentous inclusions and rimmed vacuoles within the muscle tissue [19,20].

Genetic studies identified a unique haplotype on chromosome 2p13 shared among affected individuals, which led to the discovery of a heterozygous founder mutation in the *TIA1* gene (c.1362G > A; p.E384K). This mutation causes a single amino acid substitution in the C-terminal domain of TIA1. While typically present in heterozygosity, homozygous cases are associated with more rapid disease progression [21,22]. Recent research has explored the cellular consequences of this TIA1 mutation [21,22,23,24,25,26,27,28]. The mutant variant disrupts normal SG dynamics, leading to the formation of larger, more persistent aggregates [21,23,24,25,26,27,28]. Additionally, it alters the behavior of processing bodies (PBs) and has an impact on mitochondrial dynamics, autophagy, and apoptosis [25,26,27,28]. These findings suggest a dysfunctional crosstalk between SGs and PBs, contributing to the pathogenesis of WDM by interfering with critical stress response and regulatory pathways [25,26,27,28]. Furthermore, understanding the molecular mechanisms by which the TIA1 mutation drives WDM is essential for developing effective therapies. Given TIA1′s central role, it represents a promising therapeutic target. Interestingly, the same mutation has also been linked to cases of amyotrophic lateral sclerosis (ALS), underscoring its potential relevance in other neurodegenerative diseases [18,29,30].

In this study, we examined the behavior of the WDM-associated TIA1 mutated protein compared with the wild-type (WT) protein under various cellular stress conditions, including proteotoxic, proteostatic, oxidative, and osmotic/saline stresses, as well as the degree of eukaryotic translation initiation factor 2 subunit alpha (eIF2α) Ser35 phosphorylation dependency. Our findings consistently show that TIA1 E384K (TIA1^WDM^) alters the dynamics of SG formation and disassembly in response to these stressors, with a clear trend toward deleterious effects depending on the nature of the stimulus in eIF2α-dependent phosphorylation. These results offer new insights into the role of TIA1^WDM^ in regulating SGs and the implications for disease. By elucidating these stress-specific behaviors, our work paves the way for future research into therapeutic strategies aimed at restoring normal SG dynamics in WDM and potentially other stress-related disorders.

## 2. Materials and Methods

### 2.1. Cell Culture

HEK293 Flp-In T-Rex cells (human embryonic kidney; Invitrogen, Carlsbad, CA, USA), hereafter referred to as FT293, were cultured as previously described [25,28,30,31]. Derivative lines FT293-GFP-TIA1a^WT^ and FT293-GFP-TIA1a^WDM^, expressing tetracycline-inducible GFP-tagged TIA1a fusion proteins, were generated using the Flp-In T-Rex system and maintained according to established protocols [25,28]. Cells were routinely passaged upon reaching ~90% confluence and incubated in a humidified environment at 37 °C with 5% CO_2_ and 95% air (Thermo Electron Corporation, Waltham, MA, USA).

### 2.2. Stress Induction

Expression of GFP-TIA1a^WT^ and GFP-TIA1a^WDM^ was induced by the addition of tetracycline (100 ng/mL; Merck, Darmstadt, Germany). Tetracycline induction was performed at ~50% confluence, prior to stress exposure. Stress treatments were applied 24 h post-induction, as follows: heat shock (45 °C for 60 min) in a hybridization oven/shaker (Amersham Life Sciences); proteostatic stress (1 µM thapsigargin for 90 min); oxidative stress (0.5 mM sodium arsenite [NaAsO_2_] for 60 min); and saline stress (150 mM sodium chloride [NaCl] for 45 min). For recovery experiments, stress-inducing agents were removed at the indicated times. Cells were then washed with PBS, fresh medium was added, and analysis was conducted three hours post-recovery.

### 2.3. Western Blot Analysis

Proteins were separated via 10% SDS-PAGE and transferred to nylon membranes (Merck Millipore Ltd., Darmstadt, Germany) at 4 °C and 50 V for two hours. Membranes were blocked with 5% non-fat milk in phosphate-buffered saline (PBS) containing 0.1% Tween-20 (PBS-Tween), then incubated overnight at 4 °C with primary antibodies in PBS-Tween containing 3% BSA (bovine serum albumin, Sigma, Fukushima, Japan). After washing, membranes were incubated with horseradish peroxidase (HRP)-conjugated secondary antibodies for one hour at room temperature. Detection was performed using ECL reagent (GE Healthcare, Chicago, IL, USA). The primary antibodies used were as follows: anti-TIA1 (sc-1751; 1:4000; Santa Cruz, Monterey Bay, CA, USA); anti-T-cell-related TIA1 (TIAR) (sc-1749; 1:3000; Santa Cruz; anti-total eukaryotic translation initiation factor 2 subunit alpha (eIF2α) (sc-133132; 1:4000; Santa Cruz); anti-phospho-eIF2α (S51) (9721L; 1:1000; Cell Signaling); anti-Hu antigen R (HuR) (3A2) (sc-5261; 1:3000; Santa Cruz); and anti-tubulin subunit alpha (TUBA) (T5168; 1:5000; Merck). Secondary antibodies were from goat (Santa Cruz Biotechnology, Dallas, TX, USA), rabbit, and mouse (Promega, Madison, WI, USA) as appropriate [25,28,30,31].

### 2.4. Immunofluorescence and Confocal Microscopy

FT293-GFP-TIA1a^WT^ and FT293-GFP-TIA1a^WDM^ cells were plated on poly-L-lysine-coated coverslips (Sigma-Aldrich, Burlington, MA, USA) for three to four hours. After applying the stress treatments as described, cells were fixed with 10% formalin (Merck) and processed according to established protocols [25,28]. Nuclear staining was performed using To-Pro-3 (1 µM; ThermoFisher, Waltham, MA, USA). SGs were detected using a polyclonal anti-GTPase-activating protein (SH3 domain) binding protein 1 (G3BP1) antibody (1:500; Cusabio, Houston, Texas, USA). Images were acquired using a Nikon A1R+ confocal microscope (Nikon Corporation, Tokyo, Japan) coupled to an Eclipse Ti-E inverted microscope. Image processing and analysis were performed using ImageJ 1.54k [25,28].

### 2.5. Statistical Analysis

All data are presented as mean ± standard error of the mean (SEM), and a non-paired two-sided Student’s *t*-test was used to determine statistical significance between two groups. *p*-values of < 0.05 were considered statistically significant.

## 3. Results

### 3.1. Analysis of eIF2α Phosphorylation Dynamics in FT293-TIA1a^WT/WDM^ Cells Under Distinct Stress Conditions

**Figure 1:**
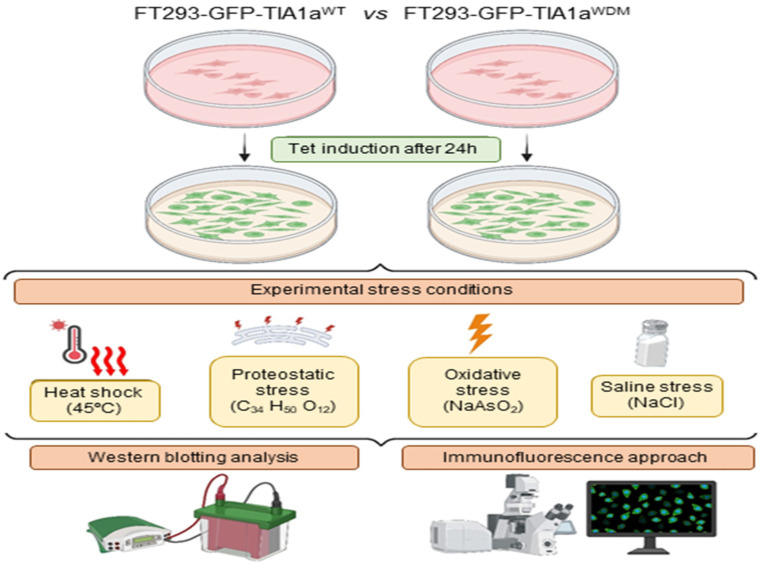
Overview of the experimental workflow. Details of wild-type (WT) and WDM-GFP-TIA1a-expressing FT293-HEK293 cells under heat shock (45 °C), proteostatic (C_34_ H_50_ O_12_/thapsigargin), oxidative (NaAsO_2_/sodium arsenite), and saline/osmotic (NaCl/sodium chloride) stress conditions, together with cellular (immunofluorescence confocal microscopy) and proteomic (Western blot) analysis to study TIA1-dependent stress granule dynamics.

To investigate the impact of TIA1^WDM^ on SG dynamics under different stress conditions, we designed an experimental study to assess the effects of proteotoxic, proteostatic, oxidative, and osmotic/saline stressors on FT293-GFP-TIA1a^WT/WDM^ cell lines (Figure 1).

**Figure 2:**
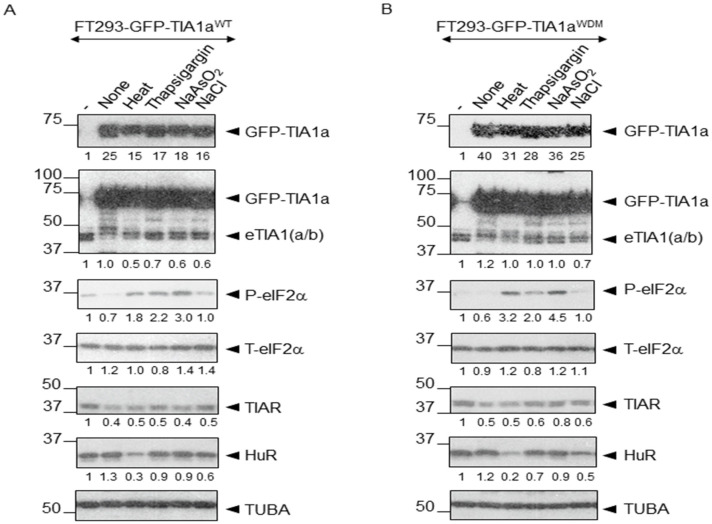
eIF2α phosphorylation-dependent dynamics of FT293-HEK293-TIA1a^WT/WDM^ cells under distinct stress conditions. (**A**,**B**) Western blot analysis of total cell extracts (15 μg) from FT293-GFP-TIA1a^WT^ (**A**) and FT293-GFP-TIA1a^WDM^ (**B**) cell subjected to the following stress treatments (as indicated in the legend): heat shock (45 °C for 60 min), thapsigargin (1 μM for 90 min), sodium arsenite (NaAsO_2_, 0.5 mM for 60 min), and sodium chloride (NaCl, 150 mM for 45 min). Blots were probed with antibodies against TIA1, phosphorylated eIF2α (P-eIF2α), total eIF2α (T-eIF2α), TIAR, HuR, and TUBA. Molecular weight markers (kDa) and protein identities are shown on the left and right, respectively. The relative quantification of the intensity of each band of the analyzed proteins was corrected in comparison with the loading control (TUBA values) and entered at the bottom of each insert. Densitometric quantification was performed using ImageJ software.

Using the HEK-Flp cell line after 24 h of tetracycline-induced expression of the GFP-TIA1a^WT^ and GFP-TIA1a^WDM^ constructs [25,28], we subjected isogenic FT293-GFP-TIA1a^WT/WDM^ cells to proteotoxic, proteostatic, chemotoxic, and osmotic stress conditions as described in Materials and Methods, Section 2.2. Next, we analyzed the GFP-TIA1^WT/WDM^-tagged expression and phosphorylated eIF2α-dependent stress induction as well as assembly and disassembly dynamics of TIA1^WT/WDM^-dependent SGs using Western blot and immunofluorescence confocal microscopy, respectively (Figure 1). Indeed, to confirm the correct expression of ectopic GFP-tagged TIA1a^WT/WDM^ fusion proteins and endogenous markers (TIA1, TIAR, HuR, and TUBA), as well as the phosphorylation status of eIF2α, we performed Western blot analysis with specific antibodies under the indicated stress conditions (Figure 2A,B).

Phosphorylation of eIF2α—a key indicator of inhibited 5’-cap-dependent translation [4,15]—was assessed to distinguish between eIF2α phosphorylation-dependent stressors (Figure 2A,B). Additionally, we have analyzed the expression of GFP-TIA1^WT/WDM^, RBPs (TIAR and HuR), and TUBA (loading control) (Figure 2A,B). These proteomic patterns were consistent with previous observations [4,15,25,26,27,28,30]. These findings also suggest that the degree of steady-state expression of WT and WDM-associated TIA1 was stressor-independent.

These observations confirmed that there was no GFP-TIA1a^WT/WDM^ expression in the absence of tetracycline, indicating a robust and leak-free induction system that remained consistent across treatments. HuR expression was unaffected by proteostatic, oxidative, or saline stresses, demonstrating its stability against these insults; however, its expression was affected by thermal stress (Figure 2A,B). In both FT293-GFP-TIA1a^WT^ and FT293-GFP-TIA1a^WDM^ cell lines, heat shock, thapsigargin, and sodium arsenite treatments significantly increased eIF2α phosphorylation. In contrast, this was significantly reduced or refractory under saline conditions, aligning with previous reports [3,4,15,32]. However, total eIF2α levels remained constant, indicating that the changes in phosphorylation were due to post-translational modifications rather than altered protein levels. Notably, FT293-GFP-TIA1a^WT^ cells exhibited baseline eIF2α phosphorylation even without tetracycline or arsenite, possibly due to some prior culture-related stress, which was absent in FT293-GFP-TIA1a^WDM^ cells.

To determine whether TIA1^WMD^ affects SG dynamics uniformly or in a stress-specific manner, we exposed FT293-GFP-TIA1a^WT/WDM^ cells to heat shock, endoplasmic reticulum (ER), oxidative, and osmotic stressors under the above conditions. We then analyzed TIA1-dependent SG dynamics using immunofluorescence confocal microscopy, combining the distribution and localization of ectopic GFP-TIA1a^WT/WDM^ and endogenous G3BP1 (an early SG nucleator), as well as nuclear staining with To-Pro-3 reagent.

### 3.2. Dynamics of TIA1-Dependent SGs Under Heat Shock

**Figure 3:**
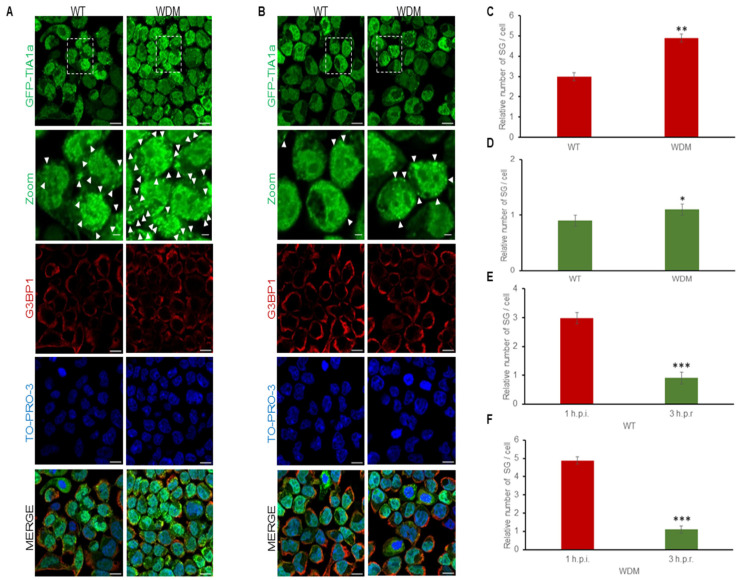
Dynamics of stress granule assembly and disassembly in FT293-HEK293 cells expressing TIA1a^WT^ and TIA1a^WDM^ under heat shock. (**A**,**B**) Immunofluorescence images display TIA1 (green) and G3BP1 (red) in FT293-GFP-TIA1a^WT^ and FT293-GFP-TIA1a^WDM^ cells during a one-hour heat shock at 45 °C (**A**) and three hours post-heat shock (**B**). The outlined boxes in GFP-TIA1a panels (**A**,**B**) are enlarged details in the zoom images. The details are a 2 x zoom images. The white arrowheads indicate TIA1-SGs. Nuclei are stained with To-pro-3; scale bar, 20 μm. (**C**–**F**) Quantifications of the relative number of stress granules (SG) and their assembly and disassembly dynamics under the conditions described above (**A**,**B**) from one-hour after induction (1 h.p.i.) (**C**) to three hours after recovery (3 h.p.r.) (**D**–**F**). Data are shown as mean ± standard error of the mean (SEM) (n = 62–308 cells). Significant differences in the relative number of SG, determined by Student’s *t*-test, are marked with (* *p* < 0.05; ** *p* < 0.001; *** *p* < 0.0001).

FT293-GFP-TIA1a^WT/WDM^ cells were treated with tetracycline for 24 h to induce GFP-TIA1a^WT/WDM^ expression, displaying the characteristic nucleocytoplasmic distribution of these variants [19,20,21,22]. After heat shock insult (45 °C, 60 min), GFP-TIA1a^WDM^ exhibited significantly enhanced localization to SGs compared with GFP-TIA1a^WT^, with an increase in small SG number rather than size, as detected by GFP-TIA1 fluorescence and co-staining with anti-G3BP1 antibody followed by immunofluorescence analysis (Figure 3A–C). In fact, the main problem is the diffuse distribution effect associated with proteotoxicity under heat shock stress, which is reinforced by the alteration of cell morphology and the small size of the generated SG in this thermal condition in the HEK-293 cell line (Figure 3A,B, zoom images).

After a three-hour recovery period in the absence of heat shock stimuli, TIA1a-SG formation was fully reversible, though disassembly was slightly slower in TIA1a^WDM^-SGs compared with TIA1a^WT^-SGs (Figure 3C–F). These results suggest that both the assembly and disassembly dynamics of TIA1a^WDM^-dependent SGs are significantly altered compared with the dynamics of TIA1a^WT^-dependent SGs under heat shock.

### 3.3. Dynamics of TIA1-Dependent SGs Under Endoplasmic Reticulum-Dependent Stress

**Figure 4:**
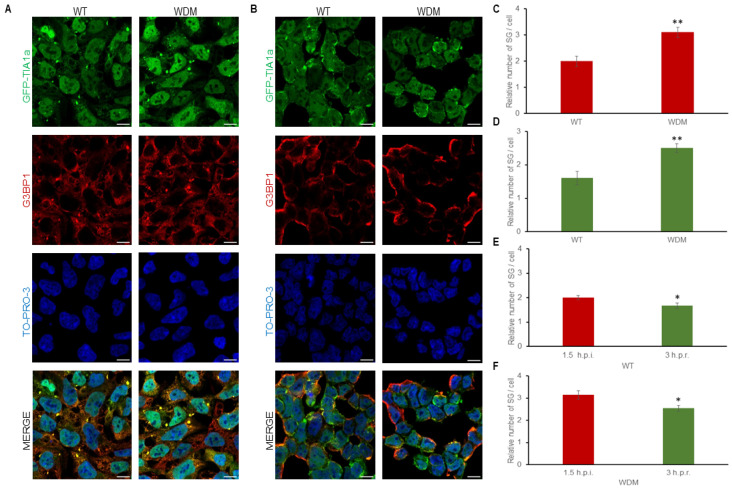
Dynamics of stress granule assembly and disassembly in FT293-HEK293 cells expressing TIA1a^WT^ or TIA1a^WDM^ under thapsigargin-induced endoplasmic reticulum stress. (**A**,**B**) Immunofluorescence images show TIA1 (green) and G3BP1 (red) in FT293-GFP-TIA1a^WT^ and FT293-GFP-TIA1a^WDM^ cells treated with 1.5 μM thapsigargin for 1.5 h (**A**) and three hours after removal of proteostatic stress (**B**). Nuclei are stained with To-pro-3; scale bar, 20 μm. (**C**–**F**) Quantifications of the relative number of stress granules (SG) and their assembly and disassembly dynamics from A and B as described in Figure 3, presented as mean ± SEM (n = 49–103 cells). Significant differences in relative number of SG, determined by Student’s *t*-test, are indicated (* *p* < 0.05; ** *p* < 0.001).

FT293-GFP-TIA1a^WT/WDM^ cells treated with thapsigargin (1 μM, 90 min), an ER-dependent/specific proteostatic stressor involving PERK kinase and eIF2α phosphorylation, were also visualized by immunofluorescence analysis (Figure 4A,B). The results showed patterns of differential TIA1-dependent SG formation in FT293-GFP-TIA1a^WT/WDM^ cells. Under these conditions, GFP-TIA1a^WDM^ showed a slight but significant increase in localization to SGs compared with GFP-TIA1a^WT^.

After a three-hour recovery period following proteostatic insult, TIA1a^WT/WDM^-SG disassembly was slower compared to that induced by heat shock and other stress conditions used in this study (Figure 4D–F). While the SG number decreased gradually, SG size was significantly reduced due to progressive fission and disaggregation of initially larger TIA1-SGs into smaller structures (Figure 4C–F). Altogether, these observations indicate that the dynamics of TIA1a^WDM^-SGs are slightly more sensitive under ER-associated stress with thapsigargin compared with TIA1a^WT^-SGs.

### 3.4. Dynamics of TIA1-Dependent SGs Under Oxidative Stress

**Figure 5:**
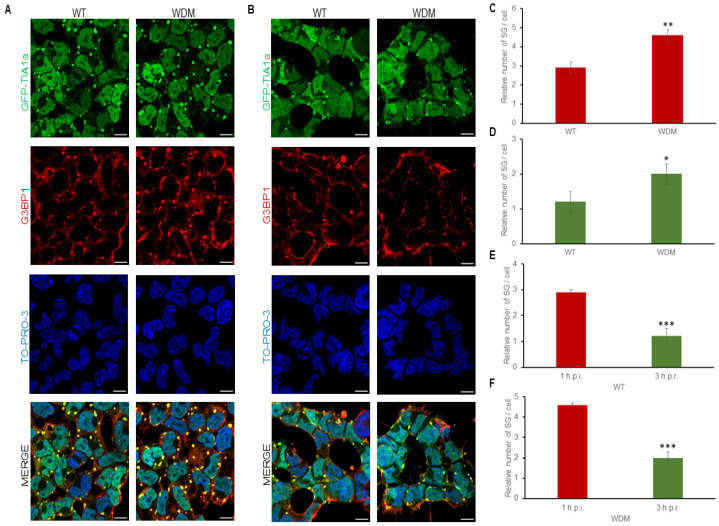
Dynamics of stress granule assembly and disassembly in FT293-HEK293 cells expressing TIA1a^WT^ or TIA1a^WDM^ under sodium arsenite-induced oxidative stress. (**A**,**B**) Immunofluorescence images display TIA1 (green) and G3BP1 (red) in FT293-GFP-TIA1a^WT^ and FT293-GFP-TIA1a^WDM^ cells treated with 0.5 mM sodium arsenite for 60 min (**A**) and three hours after the removal of oxidative stress (**B**). Nuclei are stained with To-pro-3; scale bar, 20 μm. (**C**–**F**) Quantifications of the relative number of stress granules (SG) and their assembly and disassembly dynamics from A and B as described in Figure 3, shown as mean ± SEM (n = 103–169 cells). Significant differences in relative number of SG, determined by Student’s *t*-test, are marked (* *p* < 0.05; ** *p* < 0.001; *** *p* < 0.0001).

Next, FT293-GFP-TIA1a^WT/WDM^ cells were treated with sodium arsenite (NaAsO_2_, 0.5 mM, 60 min) followed by a three-hour recovery period. SG assembly exhibited TIA1-dependent dynamics consistent with prior studies [21,23,25,26,27,28]. Oxidative stress rapidly induced the formation of small TIA1-SGs that were fused, with an increase in fusion events in FT293-GFP-TIA1^WDM^ cells, leading to larger SGs compared with FT293-GFP-TIA1^WT^ cells (Figure 5A,B).

Regarding the three-hour recovery dynamics, the delayed disassembly in FT293-GFP-TIA1a^WDM^ cells was attributed to larger SG sizes (Figure 5C), highlighting the impact of the TIA1^WDM^ mutation on TIA1a^WT^-dependent SG dynamics (Figure 5C–F). These findings replicate and reinforce the consistent effect of the WDM-TIA1 mutation on TIA1-SG dynamics under oxidative stress, as induced by sodium arsenite.

### 3.5. Dynamics of TIA1-Dependent SGs Under Osmotic Stress

**Figure 6:**
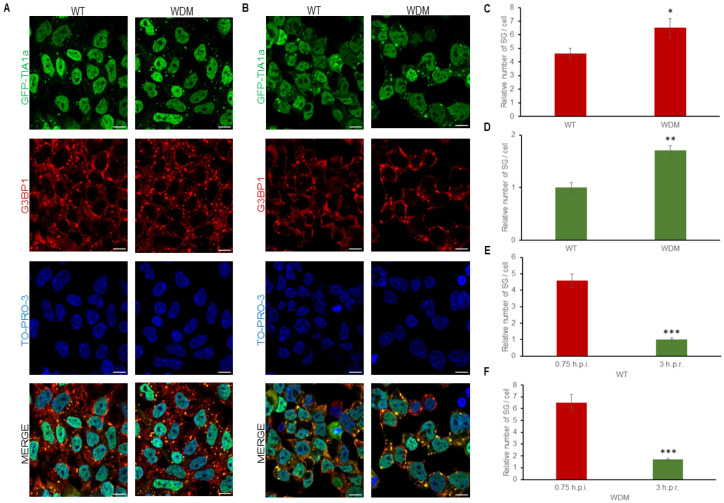
Dynamics of stress granule assembly and disassembly in FT293-HEK293 cells expressing TIA1a^WT^ or TIA1a^WDM^ under sodium chloride-induced saline stress. (**A**,**B**) Immunofluorescence images show TIA1 (green) and G3BP1 (red) in FT293-GFP-TIA1a^WT^ and FT293-GFP-TIA1a^WDM^ cells treated with complete DMEM supplemented with NaCl 150 mM for 45 min (**A**) and three hours after the removal of osmotic stress (**B**). Nuclei are stained with To-pro-3; scale bar, 20 μm. (**C**–**F**) Quantifications of the relative number of stress granules (SG) and their assembly and disassembly dynamics from A and B as described in Figure 3, presented as mean ± SEM (n = 200–314 cells). Significant differences in relative number of SG, determined by Student’s *t*-test, are indicated (* *p* < 0.05; ** *p* < 0.001; *** *p* < 0.001).

Lastly, FT293-GFP-TIA1a^WT/WDM^ cells were treated with sodium chloride (NaCl 150 mM, 60 min), an eIF2α phosphorylation-less-dependent osmotic stressor under these conditions of medium osmotic stress (Figure 2) [32]. In these experimental conditions, differential TIA1-SG formation was observed in FT293-GFP-TIA1a^WT/WDM^ cells (Figure 6A,B). Thus, TIA1a^WT/WDM^-dependent SGs were smaller than those formed under other stressors. However, there were differences both in assembly and disassembly between TIA1a^WDM^- and TIA1a^WT^-dependent SGs, in accordance with previous trends observed with the other stressors used in this study (Figure 6C–F).

However, after a three-hour recovery period, TIA1a^WT/WDM^-dependent SG disassembly was faster than under heat shock, proteostatic, or oxidative conditions, with a rapid reduction in SG number without a significant change in SG size, likely due to the faster disaggregation of smaller SGs (Figure 6C–F). These findings suggest that the dynamics of TIA1a^WT/WDM^-dependent SGs respond differentially to medium osmotic stress conditions, showing a consistent behavior associated with TIA1^WDM^.

## 4. Discussion

**Figure 7:**
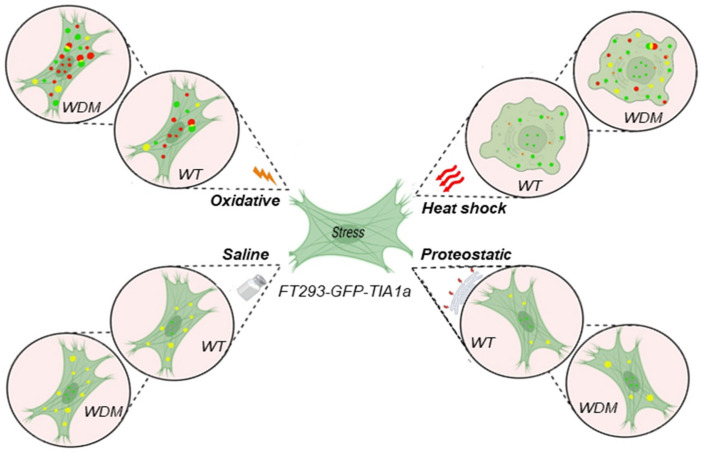
Summary of stress granule (SG) assembly and disassembly dynamics in TIA1a^WT/WDM^ cells under heat shock, proteostatic, oxidative, and saline/osmotic stress conditions. GFP-tagged TIA1^WT/WDM^ fusion proteins (green) translocated from the nucleus to the cytoplasm together with cytoplasmic nucleating actors, such as G3BP1 (red), to form SGs under the stress situations indicated. Colocalization between components is shown in yellow. This figure was created using BioRender.com (accessed on 28 January 2025).

This study investigated whether the WDM-associated TIA1 mutation differentially affects TIA1 localization into SGs under four distinct stress conditions, i.e., heat shock, ER-dependent proteostatic, oxidative, and osmotic stresses. All tested insults influenced the dynamics of WDM-associated TIA1-SGs compared with WT TIA1. These triggers/stressors induced significant TIA1 localization into G3BP1-positive SGs through eIF2α-dependent mechanisms, highlighting stressor-specific SG dynamics (Figure 7).

SGs are dynamic, non-membrane-bound cytoplasmic assemblies of RNA and proteins that form in response to cellular stress. SG composition, formation mechanisms, and dynamics vary according to cell-specific and environmental stress types [3,32,33]. Proteomic studies have identified over 400 proteins in mammalian SGs (see https://msgp.pt/ and http://rnagranuledb.lunenfeld.ca/ (accessed on 22 November 2024)) [5,7,16,24,32,33], and approximately 30–35% of cellular RNAs can be transiently locaed in SGs; however, only less than 5% can be considered as enriched [3,6]. The proteomic and transcriptomic composition of SGs varies according to cell type and stimuli, opening the possibility of the existence of subpopulations, and even selective and specific populations, depending on the cellular heterogeneity of the tissues in which they originate [3]. Thus, SGs contribute to processes of proteomic and transcriptomic remodeling involving gene expression flux, cell cycle progression, cellular dynamics, cell signaling pathways, and cell death/survival responses [3,5,7,10].

SG formation in mammalian cells is mainly initiated by the activation of the integrated stress response involving four serine/threonine kinases. These are eukaryotic translation initiation factor 2 alpha kinase 1/heme-regulated inhibitor (EIF2AK1/HRI)— which is sensitive to oxidative stress—eukaryotic translation initiation factor 2 alpha kinase 2/Protein kinase RNA-activated (EIF2AK2/PKR)—which senses viral infection—unfolded protein response (UPR)-coupled eukaryotic translation initiation factor 2 alpha kinase 3/protein kinase R-like endoplasmic reticulum kinase (EIF2AK3/PERK)—which senses UPR—and eukaryotic translation initiation factor 2 alpha kinase 4/General control non-derepressible 2 (GCN2) eIF2 alpha kinase (EIF2AK4/GCN2)—which senses amino acid starvation. These four kinases are capable of phosphorylating eIF2α at serine residue 51 [32]. This post-translational modification disassembles polyribosomes and drives translational arrest, promoting the accumulation of stalled translation preinitiation complexes (PICs) that mediate in the condensation of SGs, which involve ribosomal 40S subunits, eIFs, and polyadenylated mRNAs [4,15]. Regarding the specific stress conditions studied herein, heat shock-associated SGs, induced by elevated temperatures (42–45 °C), form as a result of disrupted protein folding and translation via phosphorylated eIF2α-dependent mechanisms. Their SGs recruit core proteins, like GTPase-activating protein (SH3 domain) binding protein 1 and 2 (G3BP1/2) and TIA1, and many other RBPs and non-RBPs. After, heat shock proteins (HSPs) are involved, aiding in protein refolding during cellular stress [6,16,24,34]. In the same vein, proteostatic stress-associated SGs arise from the accumulation of misfolded proteins due to proteasome inhibitors or chemically induced ER stress (e.g., thapsigargin). These SGs include G3BP1, TIA1, and many other RBPs and non-RBPs [4,16,32,35]. Oxidative stress-associated SGs, triggered by reactive oxygen species (ROS) and agents like sodium arsenite, contain typical SG proteins (G3BP1/2, TIA1, etc.) and persisting under severe damage potentially forming pathological aggregates [4,7,15,16,24,27,33]. Osmotic stress-associated SGs, triggered by medium and high concentrations of salt (e.g., NaCl) or osmolytes (e.g., sorbitol, sucrose, etc.), include core proteins like G3BP1, TIA1, and many others [35,36]. Across these stress types, SGs exhibit rapid assembly, dynamic fusion/fission events, and disassembly mediated by specific post-translational modifications, chaperones and helicases activities, as well as acute proteasomal and/or even autophagic responses associated with pathophysiological situations [3]. SGs and their dynamics promote distinct transcriptomic and proteomic remodeling and crosstalk with cellular pathways, processes, networks as well as another riboproteomic stable granules as P-bodies, and cellular organelles (i.e., mitochondria, lysosomes, etc.), reflecting their role as adaptive cellular triage centers [3,37].

From a historical perspective, several conceptual mechanistic phenomena have been considered and combined to explain the formation of SG in mammalian cells, each of which summarizes the progressive understanding and knowledge evolution based on the molecular composition, dynamics, and distinctive characteristics of SG. The phenomenon of “prion-like aggregation” focuses on prion-like domains located in RBPs, such as TIA1 C-terminal domain, which drive the formation of aggregation-prone SGs that can persist pathologically in neurological and muscular degenerative diseases [38,39,40,41]. The “core–shell” phenomenon suggests that SGs have a stable core of aggregated untranslated (m)RNAs and proteins, nucleated by high-affinity RBP interactions, surrounded by a dynamic liquid shell, with core persistence explaining SG heterogeneity across stressors [16,17]. The “LLPS” phenomenon posits that SGs form via weak, multivalent interactions (such as protein–protein, RNA–protein and RNA-RNA) among RBPs (G3BP1/2, TIA1, etc.), non-RBPs, mRNAs and ncRNAs, creating dynamic liquid droplets where reside stalled translation PICs. These droplets may mature into stable cores with fluid shells [42,43,44]. The “RNA-driven assembly” phenomenon prevalently highlights early RNA-RNA interactions playing a central role of G3BP1 as ‘RNA condenser’ chaperone via modulating RNA folding together with selective post-translational modifications to condense into SGs untranslated RNAs based on length and structure [6,45,46,47,48,49,50,51]. In any case, each concept/phenomenon highlights the dynamic and multiple interplay between eIFs, the components of the 40S ribosomal subunit, RBPs, non-RBPs, mRNAs, and ncRNAs [49,50,51]. In the same vein, SG disassembly in mammalian cells, critical for restoring normal function after stress, underscores dynamic and complex processes on the aforementioned mechanisms to ensure SG clearance and homeostasis recovery [52,53,54]. This process is described as a multifaceted process involving translation restoration, LLPS dissolution, RNA remodeling and protein disaggregation [55,56,57,58,59,60,61,62,63,64,65].

Defects in SG dynamics and stress sensing are common in a variety of human disorders, including but not limited to neurological/muscular degeneration, autoimmune diseases, brain ischemia, cancer, cataracts, glaucoma, diabetes, and viral infections [3,58]. The persistence of such stable SG-like aggregates causes ALS and multisystem proteinopathy, characterized by inclusions in motor neurons, brain, muscle, and bone cells [58]. For instance, hyper-phosphorylation of the microtubule-binding protein Tau causes pathological protein aggregation, the formation of neurofibrillary tangles, and neurodegeneration associated with several tauopathies [58]. Interestingly, Tau interacts with TIA1, which promotes Tau misfolding and aggregation [66,67]. The TIA1/Tau interaction induces SG assembly (with long-lived SG), cytotoxic and accelerate neurodegeneration [66,67]. Thus, a WDM and ALS neuromuscular stressing atlas could reveal transcriptome-proteome decoupling, proteostasis decline with SGs and PBs accumulation and their molecular components, both proteins and RNAs, and asynchronous/anomalous cleaning degradomes, promoting clustering unfolded proteins and/or RNAs contributing to the harmful phenotypes of ALS (and perhaps also WDM). In fact, Tia1-mediated SG promote neurodegeneration by sequestering Hsp70 mRNA in C9orf72 mice. This unrecognized role of Tia1-mediated SGs in promoting ALS pathogenesis by sequestering Hsp70 mRNA, suggesting potential therapeutic targets for ALS treatment [68]. In addition, recent results have also shown how many regulatory disordered elements in intrinsically disordered regions exert their effects by engaging core mRNA decay machinery. These findings define molecular features and biochemical pathways that explain how disordered regions control mRNA expression and shed light on broader principles within functional unstructured proteins [69].

On the other hand, SG may promote TIA1 aggregation and other components via intra-condensate demixing, a pathway triggered by its up concentration and post-translational modifications in a stressing agent-dependent way within condensates. Blocking this demixing pathway prevents anomalous TIA1 aggregation, highlighting a potential therapeutic strategy for ALS and WDM diseases. Mechanistically, intra-condensate demixing is triggered by local unfolding of the protein domains for intermolecular disulfide bond formation and by increased hydrophobic patch interactions in the C-terminal domain. Recently, it has been suggested that up-concentration inside condensates followed by intra-condensate demixing could be a general pathway for protein aggregation [70].

This study shows that stressors inducing high and low eIF2α phosphorylation lead to aberrant SG dynamics associated with TIA1^WDM^ expression. These findings also suggest that these stressors, and perhaps others, may contribute to the WDM phenotype, in both heterozygous and homozygous individuals, where the TIA1 E384K founder mutation functions as a dominant autosomal phenotype. Furthermore, repeated exposure to diverse stressors may accelerate/exacerbate the progression of the WDM phenotype, particularly in homozygous patients, throughout aging. Future research using cellular and animal models will be crucial in elucidating the pathophysiological consequences of WDM and identifying therapeutic strategies.

## 5. Conclusions

Cellular stress responses are essential for the survival or elimination of damaged cells, encompassing heat shock, UPR, oxidative damage, osmotic stress pathways, etc. These responses converge on common cell death or survival mechanisms, influenced by the initial stressor, cell type, and environmental factors. The WDM-associated TIA1 mutation significantly alters SG assembly and disassembly dynamics in HEK cells under various stress conditions, reflecting anomalous proteostatic events. These findings underscore the stressor-specific nature of SG dynamics and their relevance to WDM pathology, paving the way for further investigation into therapeutic interventions. Aberrant stress responses are linked to numerous diseases, including neurodegenerative disorders (e.g., ALS and frontotemporal lobar degeneration (FLD)), tauopathies, myopathies (e.g., WDM), viral infections, vascular diseases, and cancers, although the precise mechanisms remain unclear [1,10,41]. Understanding their molecular mechanisms could enable interventions to shift responses toward survival or death, depending on therapeutic goals. These insights offer significant potential for developing targeted treatments and advancing drug discovery.

## Data Availability

The original contributions presented in the study are included in the article/Supplementary Material; further inquiries can be directed to the corresponding author/s.

## Supplementary Materials

The following supporting information can be downloaded at: https://www.mdpi.com/article/10.3390/biology14091288/s1, Figure S1: Original Western blot analysis images.

## Abbreviations

The following abbreviations are used in this manuscript:

ALS	Amyotrophic lateral sclerosis
circRNAs	Circular RNAs
C9orf72	C9orf72-SMCR8 Complex Subunit
EIF2AK1/HRI	Eukaryotic translation initiation factor 2 alpha kinase 1/heme-regulated inhibitor
EIF2AK2/PKR	Eukaryotic translation initiation factor 2 alpha kinase 2/Protein kinase RNA-activated
EIF2AK3/PERK	Unfolded protein response-coupled eukaryotic translation initiation factor 2 alpha kinase 3/protein kinase R-like endoplasmic reticulum kinase
EIF2AK4/GCN2	Eukaryotic translation initiation factor 2 alpha kinase 4/General control non-derepressible 2 (GCN2) eIF2 alpha kinase
eIFs	Eukaryotic translation initiation factors
eIF2α	Eukaryotic translation initiation factor 2 subunit alpha
G3BP1/2	GTPase-activating protein (SH3 domain) binding protein 1 and 2
GFP	Green fluorescence protein
HEK293	Human embryonic kidney 293
HSP	Heat shock protein
HSPA4/HSP70	Heat shock protein family A (Hsp70) member 4
HuR	Hu antigen R
LLPS	Liquid–liquid phase separation
miRNAs	MicroRNAs
mRNA	Protein-coding RNAs
NaCl	Sodium chloride
NaAsO_2_	Sodium arsenite
ncRNAs	Short and long non-coding RNAs
PB	P-bodies
RBP	RNA-binding protein
ROS	Reactive oxygen species
SG	Stress granule
TIA1	T-cell intracellular antigen 1
TIAR	TIA1-related protein
TUBA	Tubulin subunit alpha
UPR	Unfolded protein response
WDM	Welander distal myopathy

## References

[1] Chua B.A., Van Der Werf I., Jamieson C., Signer R.A.J. (2020). Post-transcriptional regulation of homeostatic, stressed, and malignant stem cells. Cell Stem Cell.

[2] Nover L., Scharf K.D., Neumann D. (1983). Formation of cytoplasmic heat shock granules in tomato cell cultures and leaves. Mol. Cell. Biol..

[3] Biancon G., Busarello E., Cheng M., Halene S., Tebaldi T. (2025). Dissecting the stress granule RNA world: Dynamics, strategies, and data. RNA.

[4] Kedersha N., Cho M.R., Li W., Yacono P.W., Chen S., Gilks N., Golan D.E., Anderson P. (2000). Dynamic shuttling of TIA-1 accompanies the recruitment of mRNA to mammalian stress granules. J. Cell Biol..

[5] Protter D.S.W., Parker R. (2016). Principles and properties of stress granules. Trends Cell Biol..

[6] Khong A., Matheny T., Jain S., Mitchell S.F., Wheeler J.R., Parker R. (2017). The stress granule transcriptome reveals principles of mRNA accumulation in stress granules. Mol. Cell.

[7] Youn J.-Y., Dyakov B.J.A., Zhang J., Knight J.D.R., Vernon R.M., Forman-Kay J.D., Gingras A.-C. (2019). Properties of stress granule and P-body proteomes. Mol. Cell.

[8] Sanders D.W., Kedersha N., Lee D.S.W., Strom A.R., Drake V., Riback J.A., Bracha D., Eeftens J.M., Iwanicki A., Wang A. (2020). Competing protein-RNA interaction networks control multiphase intracellular organization. Cell.

[9] Tauber D., Tauber G., Parker R. (2020). Mechanisms and regulation of RNA condensation in RNP granule formation. Trends Biochem. Sci..

[10] Hirose T., Ninomiya K., Nakagawa S., Yamazaki T. (2023). A guide to membraneless organelles and their various roles in gene regulation. Nat. Rev. Mol. Cell Biol..

[11] Tian Q., Streuli M., Saito H., Schlossman S.F., Anderson P. (1991). A polyadenylate binding protein localized to the granules of cytolytic lymphocytes induces DNA fragmentation in target cells. Cell.

[12] López de Silanes I., Galbán S., Martindale J.L., Yang X., Mazan-Mamczarz K., Indig F.E., Falco G., Zhan M., Gorospe M. (2005). Identification and functional outcome of mRNAs associated with RNA-binding protein TIA-1. Mol. Cell. Biol..

[13] Wang Z., Kayikci M., Briese M., Zarnack K., Luscombe N.M., Rot G., Zupan B., Curk T., Ule J. (2010). iCLIP Predicts the dual splicing effects of TIA-RNA interactions. PLoS Biol..

[14] Van Nostrand E.L., Freese P., Pratt G.A., Wang X., Wei X., Xiao R., Blue S.M., Chen J.Y., Cody N.A.L., Dominguez D. (2020). A large-scale binding and functional map of human RNA-binding proteins. Nature.

[15] Kedersha N.L., Gupta M., Li W., Miller I., Anderson P. (1999). RNA-binding proteins TIA-1 and TIAR link the phosphorylation of eIF-2α to the assembly of mammalian stress granules. J. Cell Biol..

[16] Jain S., Wheeler J.R., Walters R.W., Agrawal A., Barsic A., Parker R. (2016). ATPase-modulated stress granules contain a diverse proteome and substructure. Cell.

[17] Wang J., Choi J.M., Holehouse A.S., Lee H.O., Zhang X., Jahnel M., Maaharana S., Lemaitre R., Pozniakovsky A., Drechsel D. (2018). A molecular grammar governing the driving forces for phase separation of prion-like RNA binding proteins. Cell.

[18] Fernández-Gómez A., Izquierdo J.M. (2022). The multifunctional faces of T-cell intracellular antigen 1 in health and disease. Int. J. Mol. Sci..

[19] Welander L. (1951). Myopathia distalis tarda hereditaria; 249 Examined cases in 72 pedigrees. Acta Med. Scand. Suppl..

[20] Borg K., Ählberg G., Anvret M., Edström L. (1998). Welander distal myopathy—An overview. Neuromuscul. Disord..

[21] Hackman P., Sarparanta J., Lehtinen S., Vihola A., Evilä A., Jonson P.H., Luque H., Kere J., Screen M., Chinnery P.F. (2013). Welander distal myopathy is caused by a mutation in the RNA-binding protein TIA1. Ann. Neurol..

[22] Klar J., Sobol M., Melberg A., Mäbert K., Ameur A., Johansson A.C., Feuk L., Entesarian M., Orlén H., Casar-Borota O. (2013). Welander distal myopathy caused by an ancient founder mutation in TIA1 associated with perturbed splicing. Hum. Mutat..

[23] Mackenzie I.R., Nicholson A.M., Sarkar M., Messing J., Purice M.D., Pottier C., Annu K., Baker M., Perkerson R.B., Kurti A. (2017). TIA1 mutations in amyotrophic lateral sclerosis and frontotemporal dementia promote phase separation and alter stress granule dynamics. Neuron.

[24] Markmiller S., Soltanieh S., Server K.L., Mak R., Jin W., Fang M., Luo E.C., Krach F., Yang D., Sen A. (2018). Context-dependent and disease-specific diversity in protein interactions within stress granules. Cell.

[25] Carrascoso I., Sánchez-Jiménez C., Silion E., Alcalde J., Izquierdo J.M. (2019). A heterologous cell model for studying the role of T-cell intracellular antigen 1 in Welander distal myopathy. Mol. Cell. Biol..

[26] Fernández-Gómez A., Velasco B.R., Izquierdo J.M. (2022). Dynamics of T-cell intracellular antigen 1-dependent stress granules in proteostasis and Welander distal myopathy under oxidative stress. Cells.

[27] Alcalde-Rey I., Ramos-Velasco B., Alcalde J., Izquierdo J.M. (2024). Decoding the molecular grammar of TIA1-dependent stress granules in proteostasis and Welander distal myopathy under oxidative stress. Cells.

[28] Ramos-Velasco B., Alcalde J., Izquierdo J.M. (2025). Welander distal myopathy-associated TIA1 mutation exacerbates P-body and stress granule dynamics concomitant with nucleolar stress under oxidative stress. Genes Dis..

[29] Purcell N., Manousakis G. (2024). Diverse phenotypic presentation of the Welander distal myopathy founder mutation, with myopathy and amyotrophic lateral sclerosis in the same family. J. Clin. Neuromuscul. Dis..

[30] Sánchez-Jiménez C., Ludeña M.D., Izquierdo J.M. (2015). T-cell intracellular antigens function as tumor suppressor genes. Cell Death Dis..

[31] Carrascoso I., Alcalde J., Sánchez-Jiménez C., González-Sánchez P., Izquierdo J.M. (2017). T-cell Intracellular antigens and Hu antigen R antagonistically modulate mitochondrial activity and dynamics by regulating optic atrophy 1 gene expression. Mol. Cell. Biol..

[32] Farny N.G., Kedersha N.L., Silver P.A. (2009). Metazoan stress granule assembly is mediated by P-eIF2alpha-dependent and -independent mechanisms. RNA.

[33] Aulas A., Fay M.M., Lyons S.M., Achorn C.A., Kedersha N., Anderson P., Ivanov P. (2017). Stress-specific differences in assembly and composition of stress granules and related foci. J. Cell Sci..

[34] Wippich F., Bodenmiller B., Trajkovska M.G., Wanka S., Aebersold R., Pelkmans L. (2013). Dual specificity kinase DYRK3 couples stress granule condensation/dissolution to mTORC1 signaling. Cell.

[35] Bounedjah O., Hamon L., Savarin P., Desforges B., Patrick A., Curmi P.A., Pastré D. (2012). Macromolecular crowding regulates assembly of mRNA stress granules after osmotic stress. J. Biol. Chem..

[36] Liu Z., Zhang Y., Li J. (2024). IRE1α colocalizes with stress granules to regulate XBP1 mRNA splicing during ER stress. J. Cell Biol..

[37] Lin Y., Protter D.S.W., Rosen M.K., Parker R. (2015). Formation and maturation of phase-separated liquid droplets by RNA-binding proteins. Mol. Cell.

[38] Kedersha N., Chen S., Gilks N., Li W., Miller I.J., Stahl J., Anderson P. (2002). Evidence that ternary complex (eIF2-GTP-tRNA(i)(Met))-deficient preinitiation complexes are core constituents of mammalian stress granules. Mol. Biol. Cell.

[39] Gilks N., Kedersha N., Ayodele M., Shen L., Stoecklin G., Dember L.M., Anderson P. (2004). Stress granule assembly is mediated by prion-like aggregation of TIA-1. Mol. Biol. Cell.

[40] El-Naggar A.M., Sorensen P.H. (2023). A new phase of networking: The molecular composition and regulatory dynamics of mammalian stress granules. Chem. Rev..

[41] Desai M., Gulati K., Agrawal M., Ghumra S., Sahoo P.K. (2025). Stress granules: Guardians of cellular health and triggers of disease. Neural Regen. Res..

[42] Brangwynne C.P., Eckmann C.R., Courson D.S., Rybarska A., Hoege C., Gharakhani J., Jülicher F., Hyman A.A. (2009). Germline P granules are liquid droplets that localize by controlled dissolution/condensation. Science.

[43] Molliex A., Temirov J., Lee J., Coughlin M., Kanagaraj A.P., Kim H.J., Mittag T., Taylor J.P. (2015). Phase separation by low complexity domains promotes stress granule assembly and drives pathological fibrillization. Cell.

[44] Boeynaems S., Alberti S., Fawzi N.L. (2018). Protein phase separation: A new phase in cell biology. Trends Cell Biol..

[45] Han T.W., Kato M., Xie S., Wu L.C., Mirzaei H., Pei J., Chen M., Xie Y., Allen J., Xiao G. (2012). Cell-free formation of RNA granules: Bound RNAs identify features and components of cellular assemblies. Cell.

[46] Namkoong S., Ho A., Woo Y.M., Kwak H., Lee J.H. (2018). Systematic characterization of stress-induced RNA granulation. Mol. Cell.

[47] Van Treeck B., Protter D.S.W., Matheny T., Khong A., Link C.D., Parker R. (2018). RNA self-assembly contributes to stress granule formation and defining the stress granule transcriptome. Proc. Natl. Acad. Sci. USA.

[48] Glauninger H., Hickernell C.J.W., Bard J.A.M., Drummond D.A. (2022). Stressful steps: Progress and challenges in understanding stress-induced mRNA condensation and accumulation in stress granules. Mol. Cell.

[49] Padrón A., Iwasaki S., Ingolia N.T. (2019). Proximity RNA labeling by APEX-seq reveals the organization of translation initiation complexes and repressive RNA granules. Mol. Cell.

[50] Holehouse A.S., Alberti S. (2025). Molecular determinants of condensate composition. Mol. Cell.

[51] Parker D.M., Tauber D., Parker R. (2025). G3BP1 promotes intermolecular RNA-RNA interactions during RNA condensation. Mol. Cell.

[52] Wheeler J.R., Matheny T., Jain S., Abrisch R., Parker R. (2016). Distinct stages in stress granule assembly and disassembly. Elife.

[53] Ganassi M., Mateju D., Bigi I., Mediani L., Poser I., Lee H.O., Seguin S.J., Morelli F.F., Vinet J., Leo G. (2016). A surveillance function of the HSPB8-BAG3-HSP70 chaperone complex ensures stress granule integrity and dynamism. Mol. Cell.

[54] Mateju D., Franzmann T.M., Patel A., Kopach A., Boczek E.E., Maharana S., Lee H.O., Carra S., Hyman A.A., Alberti S. (2017). An aberrant phase transition of stress granules triggered by misfolded protein and prevented by chaperone function. EMBO J..

[55] Buchan J.R., Kolaitis R.-M., Taylor J.P., Parker R. (2013). Eukaryotic stress granules are cleared by autophagy and Cdc48/VCP function. Cell.

[56] Seguin S.J., Morelli F.F., Vinet J., Amore D., De Biasi S., Poletti A., Rubinsztein D.C., Carra S. (2014). Inhibition of autophagy, lysosome and VCP function impairs stress granule assembly. Cell Death Differ..

[57] Chitiprolu M., Jagow C., Tremblay V., Bondy-Chorney E., Paris G., Savard A., Palidwor G., Barry F.A., Zinman L., Keith J. (2018). A complex of C9ORF72 and p62 uses arginine methylation to eliminate stress granules by autophagy. Nat. Commun..

[58] Wolozin B. (2012). Regulated protein aggregation: Stress granules and neurodegeneration. Mol. Neurodegener..

[59] Cui Q., Liu Z., Bai G. (2024). Friend or foe: The role of stress granule in neurodegenerative disease. Neuron.

[60] Choy M.S., Yusoff P., Lee I.C., Newton J.C., Goh C.W., Page R., Shenolikar S., Peti W. (2015). Structural and functional analysis of the GADD34:PP1 eIF2α phosphatase. Cell Rep..

[61] Kato M., Han T.W., Xie S., Shi K., Du X., Wu L.C., Mirzaei H., Goldsmith E.J., Longgood J., Pei J. (2012). Cell-free formation of RNA granules: Low complexity sequence domains form dynamic fibers within hydrogels. Cell.

[62] Hyman A.A., Weber C.A., Jülicher F. (2014). Liquid-liquid phase separation in biology. Annu. Rev. Cell. Dev. Biol..

[63] Kroschwald S., Maharana S., Simon A. (2018). Hexanediol: A chemical probe to investigate the material properties of membrane-less organelles. Matters.

[64] Yang P., Mathieu C., Kolaitis R.-M., Zhang P., Messing J., Yurtsever U., Yang Z., Wu J., Li Y., Pan Q. (2020). G3BP1 is a tunable switch that triggers phase separation to assemble stress granules. Cell.

[65] Xiong M., Chen Y., Wang S., Zhang Z., Fan D., Li J., He X., Zhang Y., Yao Y. (2025). Liquid-liquid phase separation (LLPS) in skeletal muscle: A new frontier in muscle biology. Dev. Biol..

[66] Vanderweyde T., Apicco D.J., Youmans-Kidder K., Ash P.E.A., Cook C., Lummertz da Rocha E., Jansen-West K., Frame A.A., Citro A., Leszyk J.D. (2016). Interaction of tau with the RNA-binding protein TIA1 regulates tau pathophysiology and toxicity. Cell Rep..

[67] Apicco D.J., Ash P.E.A., Maziuk B., LeBlang C., Medalla M., Al Abdullatif A., Ferragud A., Botelho E., Balance H.I., Dhawan U. (2018). Reducing the RNA binding protein TIA1 protects against tau-mediated neurodegeneration in vivo. Nat. Neurosci..

[68] Wei Y., Li D., Yang R., Liu Y., Luo X., Zhao W., Yang H., Chen Z., Shen C., Wang Y. (2025). TIA1-mediated stress granules promote neurodegeneration by sequestering HSP70 mRNA in C9orf72 mice. Brain.

[69] Lobel J.H., Ingolia N.T. (2025). Deciphering disordered regions controlling mRNA decay in high-throughput. Nature.

[70] Yan X., Kuster D., Mohanty P., Nijssen J., Pombo-García K., Rizuan A., Franzmann T.M., Sergeeva A., Passos P.M., George L. (2025). Intra-condensate demixing of TDP-43 inside stress granules generates pathological aggregates. Cell.

