# Supplementary figures and images for: Welander Distal Myopathy-Associated TIA1 E384K Mutation Disrupts Stress Granule Dynamics Under Distinct Stress Conditions
Source: Biology (Basel). 2025 Sep 18;14(9):1288. doi: 10.3390/biology14091288 (PMC12467723; doi:10.3390/biology14091288)

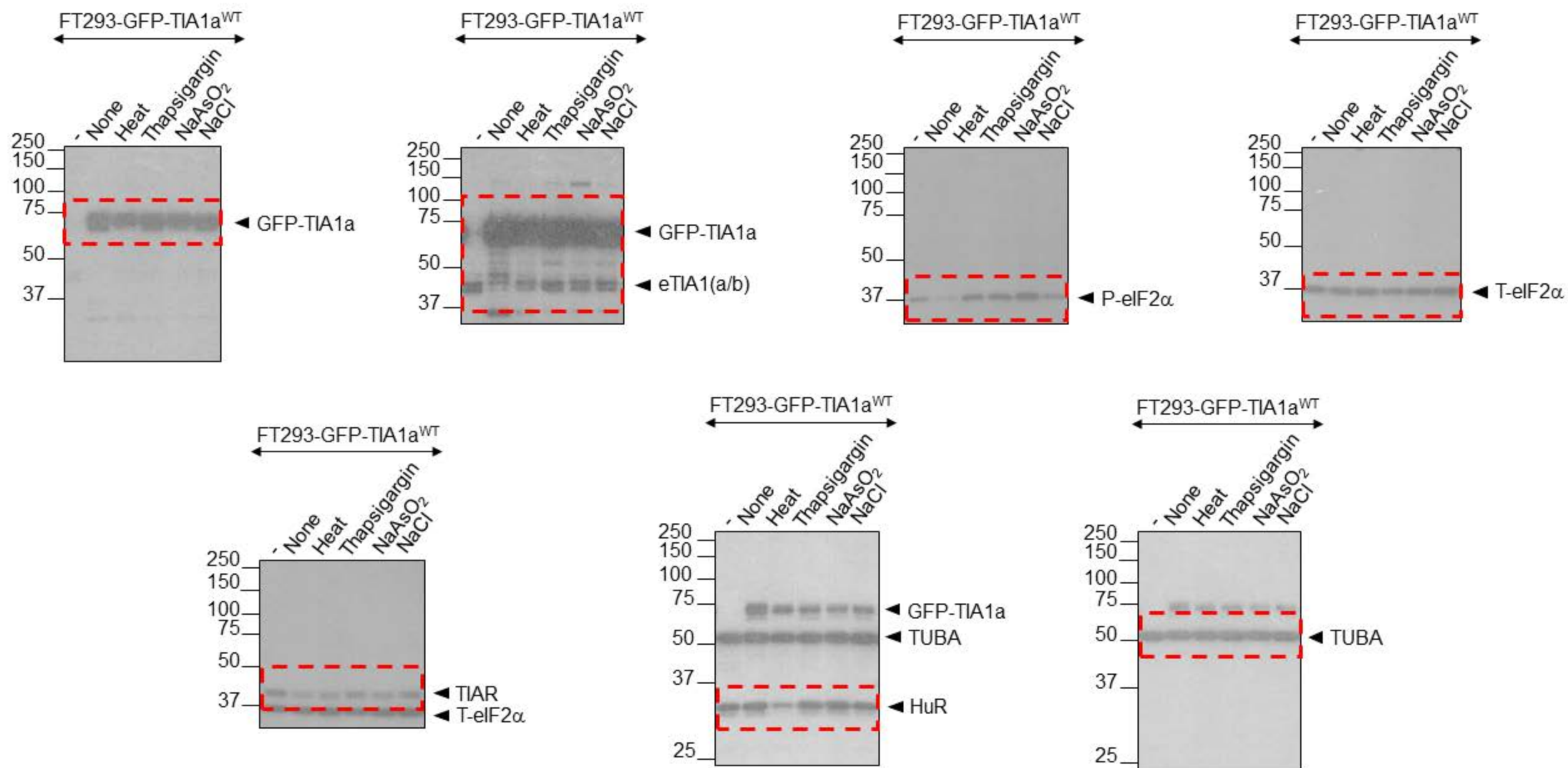

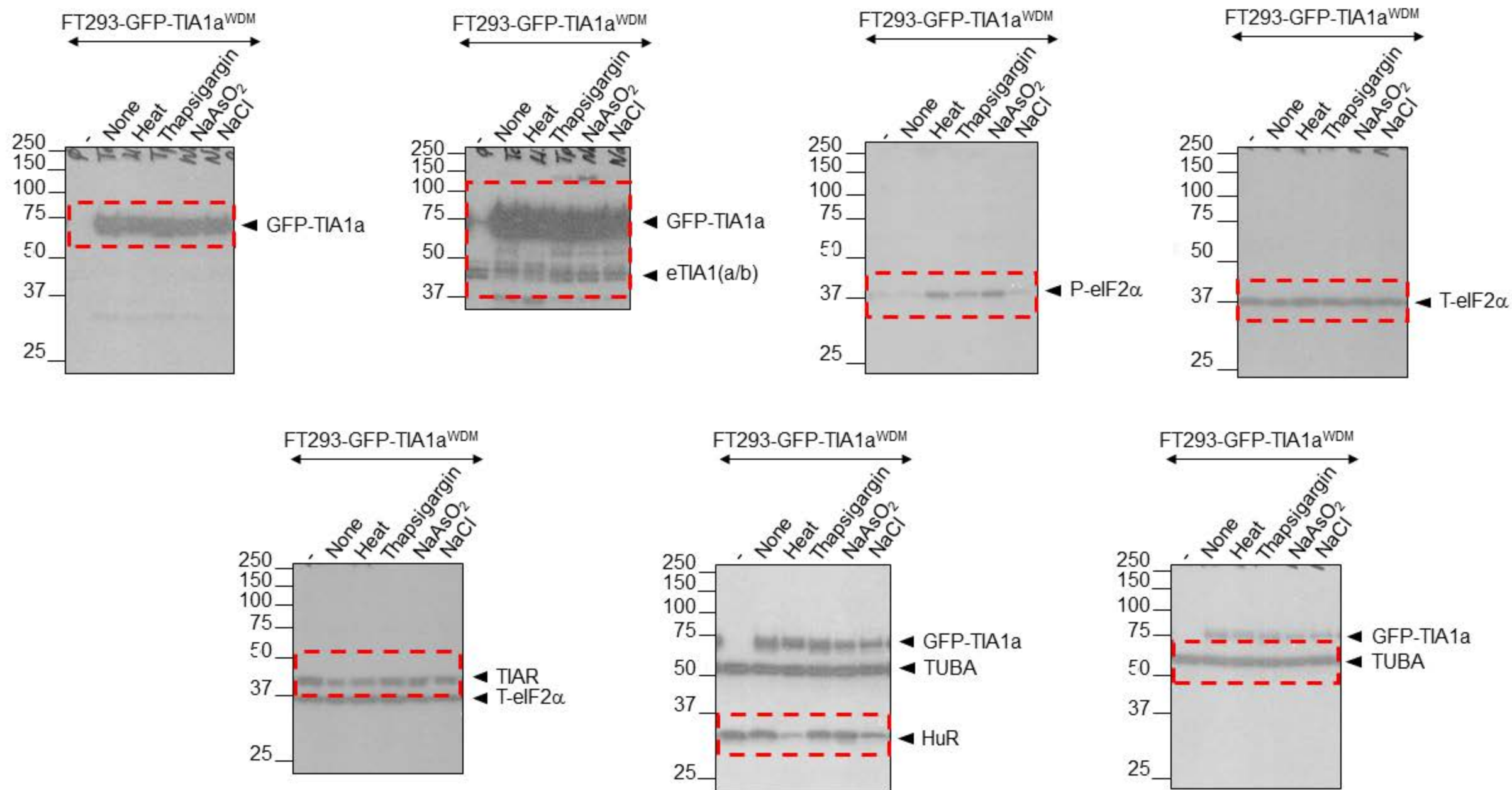

Supplement: Supplementary file 1 [file biology-14-01288-s001.zip › biology-3807694-supplementary.pdf]
